# *Ralstonia solanacearum* – A soil borne hidden enemy of plants: Research development in management strategies, their action mechanism and challenges

**DOI:** 10.3389/fpls.2023.1141902

**Published:** 2023-02-24

**Authors:** Zhaojun Wang, Wenbo Luo, Shujia Cheng, Hongjie Zhang, Jing Zong, Zhe Zhang

**Affiliations:** ^1^ State Environmental Protection Key Laboratory of Wetland Ecology and Vegetation Restoration, Changchun, China; ^2^ School of Environment, Northeast Normal University, Changchun, China; ^3^ Economy College of Changchun University, Changchun, China

**Keywords:** IDM, management, soil pathogen, plants, bacteria

## Abstract

Plant pathogens present in soil cause severe losses to plants every year. Among them, *Ralstonia solanacearum*, because of its destructive nature, is the world’s second most damaging bacterial phytopathogen. Over 310 species of plants belonging to 42 plant families are infected by this deadly pathogen. Around the world, the bacterial wilt (BW) disease causes yield losses that range from 20 to 100%. Control measures for managing this pathogen comprises several diverse approaches. Regardless of whether several control methods are developed to manage the BW disease, efficient management strategies with eco-friendly effects and the desired level of effective control is still awaited and there is need to developed effective management methods to eliminate this fetal disease in several crops under field conditions. An analysis of development in the management strategies will provide an effective way to search and develop control methods with desirable level of effectiveness. In this review, we discussed and analyzed the information reported on the development of various management strategies for the management of *R. solanacearum* along with the comprehensive presentation on action mechanism of these management strategies. We have also made an effort to summarize the challenges that make hurdle in the effective management of this deadly pathogen. The analysis of the information in this review article will assist in future implications of management strategies and help in developing effective control measures with more efficacy.

## 1 Introduction

Pathogens in the soil are a key cause of crop losses in many important plants (Elphinstone et al., 2005). *Ralstonia solanacearum*, the soil-borne bacterium that causes bacterial wilt (BW), is the world’s second most damaging phytopathogen (Mansfield et al., 2012). Ralstonia spp. are Gram-negative, oxidaseand catalase-positive, aerobic, bacilli, thriving in soil and water (Lampropoulos et al., 2021). This plant disease is among the most damaging disease of several agriculturally important crop plants such as eggplant, tomato, pepper, potato, ginger that affects the quality and quantity of crops globally (Cai et al., 2021). Because of its high destructive nature, *R. solanacearum* is currently one of the most intensively studied plant pathogen.

Over 310 species of plants belonging to 42 plant families are infected by *Ralstonia* (Genin, 2010; Genin and Denny, 2011; Paudel et al., 2020). *Ralstonia solanacearum* Species Complex, or RSSC, is the name given to the pathogen, which is made up of three distinct species: *Ralstonia pseudosolanacearum*, *R. solanacearum*, and *R. sygzii*, having same core genome. Since the first two species have been the most thoroughly studied causes of wilt disease, we will refer to strains of these species as *Ralstonia* in this article. Although the virulence nature of the pathogen is also reported in cooler temperatures, the pathogen thrives mostly in humid and hot climates. During high infection, *Ralstonia* population can reach 10^3^–10^6^ and 10^8^ cfu/gram of soil and of plant tissue, respectively. The bacterium can remain viable for years in water or soil (Alvarez et al., 2010).

*Ralstonia* typically colonizes the root xylem tissues and infect the roots of both susceptible and resistant plants through small wounds following quick move to stem tissues. The complex water-transporting tissue, xylem is made up of a variety of cell types, both dead (tracheids) and living (parenchyma). Exopolysaccharide, a substance produced by *Ralstonia*, causes blockage in xylem that lead to the appearance of wilting in plants (Ingel et al., 2021). Bacteria move from plant roots to soil when plants wilt. In various infection states, variety of virulence factors are triggered by *Ralstonia* to promote disease (de Pedro-Jové et al., 2021). These comprise exopolysaccharide, genes that aid in movement and swimming, T-3 secreted effector, and enzymes secretions like DNAases, cell wall-degrading enzymes, and enzymes that detoxify reactive oxygen species. The diversity of the species complex, in which individual strains differ not only in host range but also in traits related to virulence and physiology, is likely the cause of pathogen’s wide host range.

Around the world, the BW pathogen causes damages about 20-60%. The bacterium severely affects the tomato crop in Uganda, resulting in 88% yield losses, and BW in pepper crop was prevalent in Ethiopia at about 100% disease incidence. BW in potato is the second-most significant disease after late blight by *Phytophthora infestans*, and losses can range from 45 to 80% (Felix et al., 2010). *R. solanacearum* is reported to affect potatoes in 1.6 MH fields in 78 countries, causing an annual loss of $848 million (Charkowski et al., 2020).

**Figure 1:**
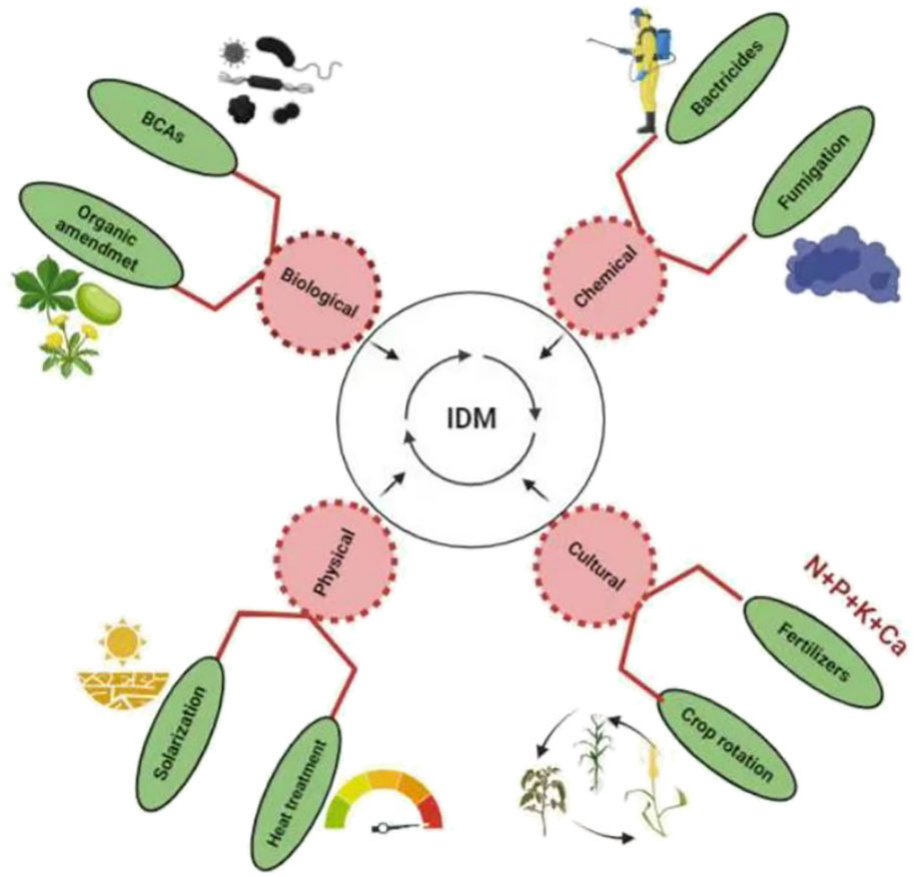
Different management strategies used for the management of *R. solanacearum*.

Control measures for managing this pathogen comprises several diverse approaches. Regardless of whether several control methods are developed to manage the BW disease, efficient management strategies with eco-friendly effects and the desired level of effective control is still awaited and there is need to developed effective management methods to eliminate this fetal disease in several crops under field conditions. An analysis of development in the management strategies will provide an effective way to search and develop control methods with desirable level of effectiveness. Here, we discussed and analyzed the information reported on the development of various management strategies for the management of *R. solanacearum* along with the comprehensive presentation on action mechanism of these management strategies (**Figure 1**). We have also made an effort to summarize the challenges that make hurdle in the effective management of this deadly pathogen.

## 2 Biological approaches

**Figure 2:**
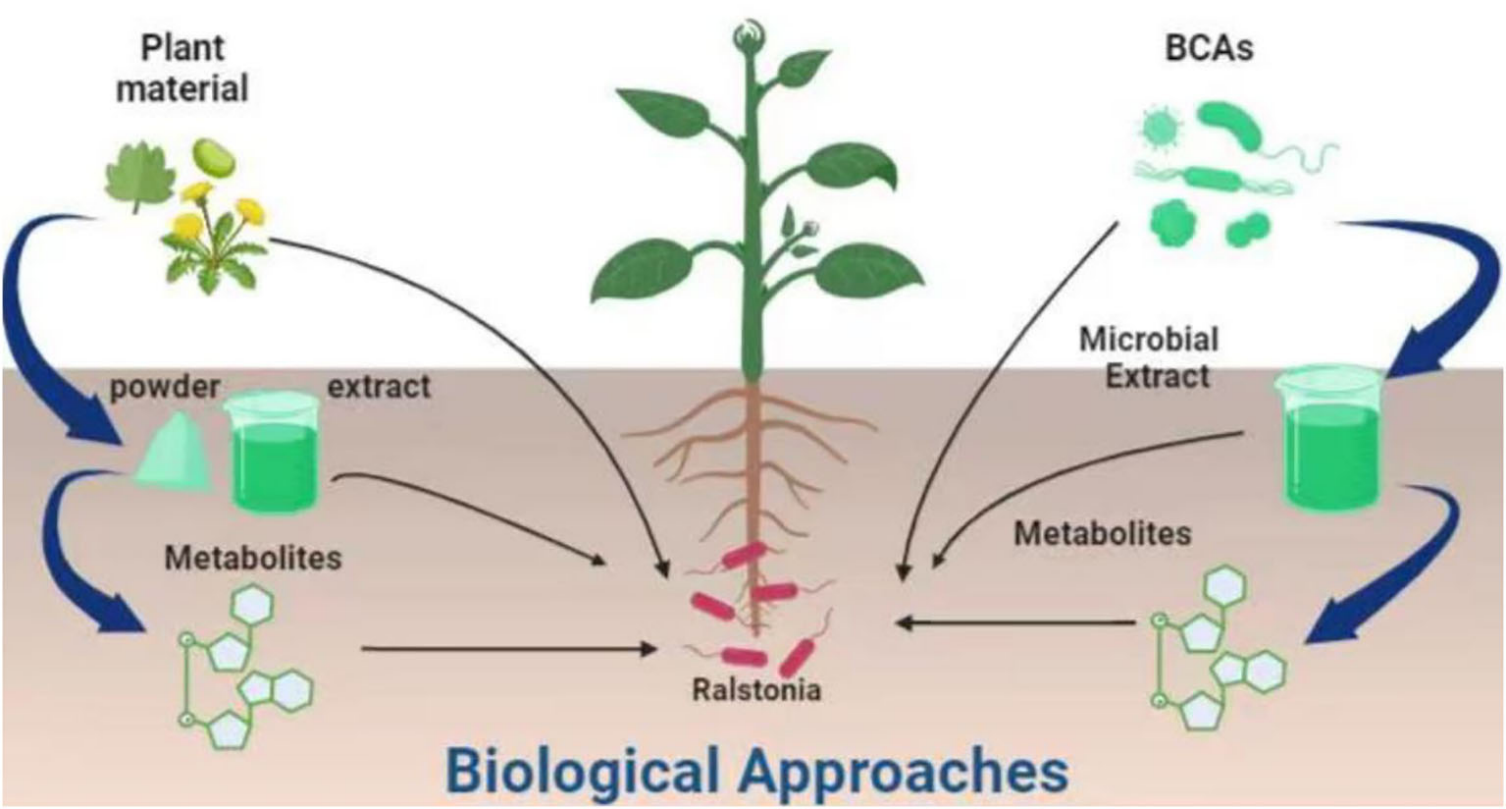
Biological approaches used for the management of *R. solanacearum*. IDM, Integrated Disease Management.

Biological approaches are those which employ natural agents, enemies, agents, or bio-based (animal, plant or microbial) products to control the pests (**Figure 2**). The ultimate aim of biological approaches is to reduce or eliminate the use of pesticides while controlling plant pests. Biological approaches are selected on the type of host, environmental conditions and target pest and its life cycle pattern. For the control of *R. solanacearum*, two general types of biological approaches i.e. the use of biocontrol agents and animal/plant base organic products were reported. Below is the detail description of these two approaches.

### 2.1 Biocontrol agents

Several beneficial characteristics that biological control agents possess have led to an increase in their use as opposed to chemical control approaches. The long-term disease suppression effect, reduction in input cost of nonrenewable resources and the self-sustaining ability are the key such features (Whipps, 2001; Wang et al., 2021). Due to the lack of effective biocontrol agents, the use of biocontrol microbes to manage bacterial wilt is less common. However, numerous reports have examined the antibacterial potential of various microbes, including bacteria, fungi, and bacteriophages, against *R. solanacearum*. Recently reported biocontrol agents and their action mechanisms are presented in **Table 1**.

**Table 1:** Recently reported biocontrol agents and their action mechanisms against *R. solanacearum* and suppression of bacterial wilt disease in various plants.

BCA	Strain No.	Host	Action mechanism	Reference
*B. velezensis*	FJAT-46737	*In vitro*	Lipopeptides production	Chen et al., 2020
*P. aeruginosa*,	—	Tomato	Lipase, protease and α-amylase enzymes	Mohammed et al., 2020
*P. syringae*	—	Tomato	Lipase, protease and α-amylase enzymes production	Mohammed et al., 2020
*P. fluorescens*	—	Tomato	Lipase, protease and α-amylase enzymes production	Mohammed et al., 2020
*Bacillus* sp.	G1S3 G4L1	Tomato	Induction of salicylic acid-, jasmonic acid-, and ethylene-dependent defenses	Fu et al., 2020
*Bacillus methylotrophicus*	DR-08	Tomato	Difficidin and oxydifficidin production	Im et al., 2020
*Paenibacillus polymyxa*	IMA5	Eggplant	Lipopeptide production	Abd Alamer et al., 2020
*Trichoderma harzianum*	—	Tomato	Metabolites	Im et al., 2020
Black Soldier Fly Pupal	—	Tomato	Chitin and Chitosan production	Kemboi et al., 2022
Bacteriophages	—	Tomato	—	Thapa Magar et al., 2022
*Bacillus velezensis*	YYC	Tomato	Host resistance	Yan et al., 2022
*Myxococcus xanthus*	R31	Tomato	secreted proteins	Dong et al., 2022
*Bradyrhizobium japonicum*	—	Tomato	Abscisic acid-production	Chattopadhyay et al., 2022
Streptomyces	—	Solanum melongena	Induces host resistance	Kaari et al., 2022
*B. amyoliquefaciens*	ZM9	Tobacco	Regulate soil physicochemical properties, promoting beneficial bacteria and antagonistic bacteria of rhizopshere soil	Hu et al., 2021

#### 2.1.1 Bacteria

According to earlier studies, bacteria predominated BCAs (90%), followed by fungi (10%). Researchers have looked into the potential efficacy of several potential bacterial BCAs. The endophytic bacterial isolate of *Enterobacter cloacae* from potato plant showed 26.5% bacterial wilt disease suppression. *Paenibacillus polymyxa*, a biocontrol bacterium in potato soil, caused 80% reduction in bacterial wilt. Several researchers have reported that endophytic and rhizobacteria belong to *Bacillus*, *Paenibacillus*, *Pseudomonas*, and *Serratia* spp. are potent BCAs against BW. A significant reduction in chilli bacterial (86%) wilt was achieved by the application of seed endospheric bacteria *Bacillus subtilis* of chili variety Firingi (Dowarah et al., 2021). In another study, two *Pseudomonas* species *P. aeruginosa* and *P. syringae* managed bacterial wilt in tomato through host resistance and antibiosis (Mohammed et al., 2020). Two endophytic isolates *Bacillus velezensis* and *Staphylococcus warneri*, among 40 that were obtained from *Gnetum gnemon* plant exhibited antibiosis activity against *R. solanacearum* and promote plant growth in tomato plants under BW stress (Agarwal et al., 2020). Yang et al. (2012) obtained a large number (420) of bacterial strains and 19 strains exhibited biocontrol effect in ginger crop. *Bacillus cereus* AR156, a forest rhizospheric soil showed 62.2% biocontrol effect against tomato BW (Wang et al., 2019). In tobacco plants, bacterial wilt was managed through the application of antagonistic *Bacillus amyloliquefaciens* strain ZM9 obtained rhizosphere of tobacco plant. Field applications of biocontrol bacteria in some cases also gave encouraging results regarding the suppression of bacterial wilt disease. Two strains and *Pseudomonas* sp. Y8 and *B*. *amyloliquefaciens* Y4 caused 3-4-fold reduction in tobacco bacterial wilt disease in field application. *B. amyloliquefaciens* FZB42 and *Bacillus artrophaeus* LSSC22 produced volatile compounds (VCs) against *R. solanacearum* (Tahir et al., 2017).

Screening of 109 bacterial isolates for their antibacterial effect against *R. solanacearum* showed that 18 active strains were belong to *Pseudomonas* spp. and 2 were belong to *Bacillus* sp. Kurabachew and Wydra (2013) obtained 13 active isolates among 150 rhizobacterial isolates that were belong to *Bacillus* spp., *Serratia marcescens*, and *Pseudomonas* spp. Studies related to *B. amyloliquefaciens* utilization as biocontrol agent against *R. solanacearum* are becoming more common increasing (Chen et al., 2020; Ding et al., 2013). The antibacterial potential of actinobacteria against *R. solanacearum* is also evident from various studies. *Actinobacteria* were reported to have several action mechanisms including induction of host resistance, production of extracellular degrading enzymes and siderophore (Di Francesco et al., 2016). Five endophytic bacteria isolated from ginger rhizomes i.e. *Acinetobacter calcoaceticus*, *P. aeruginosa*, *Enterobacter* sp., *Stenotrophomonas maltophila*, and *Klebsiella* sp. showed significant *in planta* inhibitory activity against bacterial wilt disease.

A recent development in the biocontrol management of plant diseases is the use of effective apoplastic microbes of plants. Using centrifugation and vacuum infiltration, isolated 87 isolates from ginger apoplastic fluid and tested their biocontrol effect against *R. pseudosolanacearum*. The active isolates were belonging to Enterobacteriaceae, Bacillaceae, Staphylococcaceae, and Pseudomonadaceae families. Through soil drenching and seed priming application methods, *Bacillus licheniformis* was found to have a maximum effective strain that reduced 67% disease reduction. Evaluation *in planta* during pathogenic inoculation also revealed a 71% disease decrease. Following a field test that involved solarization of soil and *B. licheniformis* application, the population of *R. pseudosolanacearum* was significantly reduced, dropping from 10^8^ to 10^3^. The disease was completely suppressed after additional field testing in farmer plots in endemic bacterial wilt regions. This integrated approach, which combines solarization technique and application of *B. licheniformis*, is a successful method of controlling ginger bacterial wilt. The bacterium is currently being advertised as a potential treatment for ginger’s bacterial wilt, and the product is called “Bacillich” (Suseela Bhai et al., 2019).

#### 2.1.2 Fungi

Aside from bacteria, some fungal strains have been reported to have a biocontrol effect on *R. solanacearum*. On the basis of findings from *in vitro* and greenhouse testing, it was reported that *Trichoderma viridae* has antibacterial potential against *R. pseudosolanacearum*. Following *Glomus versiforme* inoculation, *R. solanacearum*population on root surface as well as in the rhizosphere and xylem of tomato plants significantly reduced. Both *R. solanacearum* and *Glomus versiforme* colonization of plants caused higher concentration of roots phenols, that may have resulted in the induction of host resistance. In another study, the fungus, *Pythium oligandrum* was shown to have the biocontrol potential to suppress bacterial wilt disease and the induction of host resistance to *R. solanacearum*was attributed to cell wall proteins along with the regulation of signaling pathway for ethylene (Hase et al., 2006). *In vitro* growth of *R. solanacearum* was found to be inhibited by secondary metabolites from Trichoderma spp. (Khan et al., 2020a). Moreover, a lichen fungus *Parmotrema tinctorum* (Gomes et al., 2003) and three other fungi *Scutellospora* sp., *G. margarita*, and *G. mosseae* have been recognized as potent biocontrol fungi against bacterial wilt disease (Tahat et al., 2012).

#### 2.1.3 Bacteriophages

Bacteriophages have been reported for their biocontrol effect against BW. The biocontrol potential of bacteriophages was evaluated for the management of tobacco bacterial wilt. The host range was limited. Ramírez et al. (2020) reported the control of *R. solanacearum* in banana through lytic bacteriophages. These phages were found banana cultivated soil. Recently, a waterborne phage was succefully used to control *R. solanacearum* in irrigation water (Álvarez et al., 2019). Researchers are now investigating molecular mechanism used by phages for the biocontrol of *R. solanacearum*. Biosca et al. (2021) conducted genomic analysis of the phage having depolymerase activity. These genomic data will contribute to a better understanding of the abilities of these phages to damage host cells and, consequently, to an improvement in the biological control of *R. solanacearum*. Yamada et al., 2007 isolated several different phages and characterize them on the basis of infection they caused to specific biovar or strain of the *R. solanacearum*. Rhizosphere bacteriophages that infect *R. pseudosolanacearum* collected from ginger soil were tested for their host specificity and discovered that the isolated bacteriophage was active against the pathogen of same geographical area from which it was isolated (Yamada et al., 2007).

### 2.2 Organic approaches

Organic matter is made up of recently living organisms and decomposes or results from decomposition. It is divided into simple organic carbons or of animal and plant origin. Crop productivity has been positively impacted by the use of organic matter to manage *R. solanacearum* by improving the soil physical, chemical, and biological characteristics, which favorably affects growth of plant (Bailey and Lazarovits, 2003; Ahmad et al., 2022). Different OM, including animal waste (10%), plant residue (80%), and simple OM (10%), were reported to have suppression effect against bacterial wilt. Information about the suppression of bacterial wilt disease by plant residues are summarized in **Table 2**. Several studies showed that organic matter could effectively combat bacterial wilt in field and greenhouses. For instance, in a greenhouse evaluation, fresh parts of crotalaria and pigeon pea were used; 45 days later, they caused 100% suppression in bacterial wilt in tomato (Cardoso et al., 2006); However, because of its high application dose, this OM is not practical in field.

**Table 2:** Plants tested for their antibacterial potential against *R. solanacearum* and management of BW disease in different hosts.

Scientific name	Common name	Family	Plant part used	Host	Reference
*Psidium guajava*	Lemon guava	Myrtaceae	Leaves	*In vitro*	Acharya and Srivasta, 2009
*Piper guineense*	Benin pepper	Piperaceae	Leaves	*In vitro*	Acharya and Srivastava, 2009
*Tagetes patula*	French marigold	Asteraceae	Leaves	*In vitro*	Din et al., 2016
*Calotropis procera*	Giant milkweed	Apocynaceae	Leaves	*In vitro*	Din et al., 2016
*Adhatoda vasica*	Malabar nut	Acanthaceae	—	*In vitro*	Din et al., 2016
*Xanthium strumarium*	Rough cocklebur	Asteraceae	Shoot	Tomato	Khan et al., 2020b
*Allium sativum*	Garlic	Amaryllidaceae	—	Tomato	Abo-Elyousr and Asran, 2009
*Datura*	Datura	Solanaceae	Leaves	*In vitro*	Abo-Elyousr and Asran, 2009
*Nerium oleander*	Oleander	Apocynaceae	Shoots	*In vitro*	Abo-Elyousr and Asran, 2009
*Amanita phalloides*	Mushroom	Amanitaceae	Shoots	Potato	Erjavec et al., 2016
*Clitocybe geotropa*	Trooping funnel	Tricholomataceae	Seeds	Potato	Erjavec et al., 2016
*Hibsicus sabdariffa*	Roselle	Malvaceae	—	Potato	Hassan et al., 2009
*Punica granatum*	Pomegranate	Lythraceae	Leaves	Potato	Hassan et al., 2009
*Eucalyptus globulus*	Blue gum	Myrtaceae	—	Potato	Hassan et al., 2009
*Syringa oblata*	Broadleaf lilac	Oleaceae	Stem	Tobacco	Bai et al., 2016
*Punica granatum*	Pomegranate	Lythraceae	—	Potato	Farag et al., 2015
*Acacia*	Wattles	Fabaceae	—	Potato	Farag et al., 2015
*Eichhorina crassipes*	Water hyacinth	Pontederiaceae	Leaves	Tomato	Derib et al., 2013
*Mimosa diplotricha*	Nila grass	Fabaceae	—	*In vitro*	Derib et al., 2013
*Lantana camara*	Lantana	Verbenaceae	—	*In vitro*	Derib et al., 2013
*Prosopis juliflora*	Mesquite	Fabaceae	Leaves	*In vitro*	Derib et al., 2013
*Burcea antidysenterica*	Burcea	Burcea	Leaves	hot pepper	Yihune and Yemata, 2019
*Eucalyptus citriodora*	Lemon gum	Myrtaceae	Leaves	hot pepper	Yihune and Yemata, 2019
*Justicia schimperiana*	Agewgna	Acanthaceae	Leaves	hot pepper	Yihune and Yemata, 2019
*Lantana camara*	Lantana	Verbenaceae	Leaves	hot pepper	Yihune and Yemata, 2019
*Melia azedarach*	Chinaberry	Meliaceae	Leaves	hot pepper	Yihune and Yemata, 2019
*Ricinus communis*	Castor bean	Euphorbiaceae	Leaves	hot pepper	Yihune and Yemata, 2019
*Curcuma amada*	Mango ginger	Zingiberaceae	rhizome	*In vitro*	Karthika et al., 2017
*Ocimum gratissimum*	Clove basil	Lamiaceae	—	Tomato	Kumar et al., 2017
*Tylophora asthmatica*	Antamool	Apocynaceae	Leaves	Tomato	Kumar et al., 2017
*Calotropis gigantea*	Crown flower	Apocynaceae	—	Tomato	Kumar et al., 2017
*Ocimum sanctum*	Holy basil	Lamiaceae	Leaves	Tomato	Kumar et al., 2017
*Tylophora asthmatica*	Indian ipecacuan	Asclepiadaceae	Leaves	Tomato	Kumar et al., 2017
*Nigella sativa*	Black cumin	Ranunculaceae	Shoot	Tomato	Kumar et al., 2017
*Ruta graveolens*	Rue	Rutaceae	—	Tomato	Kumar et al., 2017

Plant base products in various forms such as green manure, dried powder and plant base oil were effectively reported to control *R. solanacearum* in infested soil. Considerable suppression in soil pathogen count (race 4) was reported by the application of essential oils from palmarosa and lemongrass. But given the high cost involved, using these oils in the field is not practical. Therefore, it is necessary to conduct field research before burying fresh crop manure (Nelson, 2013). Pathogen count have been found to be decreased and plant growth have been improved by the application of a number of medicinal plants. With the help of green manure made from various *Cajanus cajan* and *Crotalaria juncea* parts, tomato bacterial wilt was successfully controlled (Cardoso et al., 2006). Din et al., 2016 suggested that soil application with dried powder from *Adhatoda vasica, Tagetes patula*, and *Calotropis procera*, could be used as an effective management measure against bacterial wilt of tomato.

Management of plant diseases by using animal wastes is evident from many studies and some of them also suggested the suppression of bacterial wilt disease by this method. Pig slurry, for example, suppressed the pathogen count in soil. Poultry and farmyard manure in another study suppressed bacterial wilt by increasing microbial activity and increasing the population of cultural fungi and bacteria (Islam and Toyota, 2004). Pathogen’s poor survival was also linked to the reduction in disease index. Although, organic base management approaches gave encouraging results, however, there are drawbacks to their widespread use. Major determinants of organic matter’s efficacy in the suppression of plant pathogens are application rate, amendment type, decomposition stage and host-pathogen interaction (Janvier et al., 2007).

In pot experiments, the effects of simple organic substances, such as sugars on tomato BW were assessed. Lysine was added to soil and a pumice culture medium to reduce bacterial wilt in tomatoes by 70-90%, and 62-90%, respectively. Riboflavin, on the other hand, caused a number of reactions related to plant immunity and metabolic reactions in pathogen reduction, defending host plant. In tomato plants, aminobutyric acid increased polyphenol oxidase activity while decreasing catalase activity, suggesting the induction of bacterial wilt resistance. A different investigation revealed that methyl gallate had potent bactericidal effects on *R. solanacearum* (Fan et al., 2014).

## 3 Breeding and genetic engineering approaches

The most efficient, economical, and pollution free approach of pathogen management is to cultivate the highly resistant cultivars against bacterial wilt (Yuliar et al., 2015). Several important crop plants including vegetables and field crops are subjected to breeding for resistance against bacterial wilt disease. Factors that frequently had an impact on this management strategy include the diversity and availability sources for resistance, the genetic relationships between agronomic and resistance traits, host pathogen interaction mechanism (Elphinstone et al., 2005). By electrically fusing mesophyll protoplasts, somatic hybrids of *Solanum melongena* and two varieties of *Solanum aethiopicum* were created, and it was discovered that they were resistant to *R. solanacearum*. By introducing genotype CF6 of potato to phureja, bacterial wilt severity has been reduced by 90–100%. Resistant plants even severely invaded by *R. solanacearum* exhibited no wilt symptoms. It is reported limited pathogen movement from the protoxylem to other xylem tissues prevented bacterial multiplication in the stems of resistant tomato plants. The correlation between yield and quality of many crops and resistance to bacterial wilt has typically been negative. Due to other agronomic traits and the potential for poor release, resistant cultivars may not be well-received by farmers or consumers. In the future, it is anticipated that greater efforts will be made to genetically enhance bacterial wilt resistance using biotechnology approaches in order to increase crop yield.

Tomato variety with the *NPR1* gene from Arabidopsis showed significantly reduced bacterial wilt. (Lin et al., 2004). By inducing systemic acquired resistance and induced systemic resistance, the *NPR1* gene plays a crucial part in the reaction of host to pathogen infection. It also serves as the main key in the facilitation of cross-talk between jasmonic acid and salicylic acid responses. *NPR1* expression in *Arabidopsis thaliana* ensures a quick reaction to salicylic acid (SA) (Cao et al., 1998). *R. solanacearum*-infected resistant plants showed vascular tissues’ tolerance to the BW. Even though cultivar resistance has demonstrated excellent qualities in managing bacterial wilt, acceptance from public is required before such genetically modified crops can be used commercially. Additionally, the quality and quantity of the crops have mostly been inversely correlated with the reduction of BW in many cases (Yuliar et al., 2015).

Although breeding for resistance is a key disease management tactic, some crops that are severely affected by bacterial wilt disease still lack resistant varieties. For example, none of the ginger cultivars that have been made available are resistant to BW. Since ginger is a crop that is vegetatively propagated, this may be because there is little genetic variation among the accessions (Prasath et al., 2011). Among several varieties that were tested for their response to BW infection, and it was discovered *Curcuma amada* was highly resistant. By using gamma rays to induce mutations in ginger, Prasath et al. (2011) were able to create mutants that were resistant to bacterial wilt. These mutants are currently being tested in the field.

## 4 Physical approaches

Variety of physical control methods were reported for have good control effect against bacterial wilt disease. These techniques include soil disinfection, hot water treatment, and soil solarization (Yuliar et al., 2015). This section comprises the detail discussion of a number of physical methods in which *R. solanacearum* has been successfully controlled.

The selection of planting and propagating material free from infection must be given the utmost importance in order to stop field outbreaks of BW disease (Kumar and Hayward, 2005). Prior to planting, 30 days’ soil solarization was recommended to lower the soil pathogen inoculum resulting in the reduction of disease severity and enhancement in yield and seed germination (Kumar and Hayward, 2005). As a hydrothermal process, soil solarization eliminates the majority of harmful organisms, including the weeds seed without leaving any toxic residues behind. Several physical approaches were reported for soil solarization. Trapping sunlight for increasing soil temperature is one of the useful techniques for soil solarization (Kumar and Hayward, 2005). It is suggested the covering of soil with a plastic cover during protracted periods of high temperature. This aids in capturing the sun’s energy to warm the soil which suppress the pathogen population in soil. It is reported that exposing the soil to sunlight suppressed tomato BW significantly. Another study found that sterilizing ginger seeds with microwaving at 42°C completely controlled the BW disease. Before tomato plants were planted, the infected soil was subjected to heat treatment at 52°C for 3 days which resulted in the reduction of bacterial population by 50-86%. Seed pathogens can also be killed using heat treatment. After being exposed to hot air for 28 minutes, ginger seeds that are already infected by the pathogen, produced rhizomes free from infection, and the procedure had no negative effects on growth and sprouting (Kumar and Hayward, 2005).

Cold temperature treatments can occasionally be as effective as those using heat. Due to the low temperature in Queensland, *R. solanacearum* rarely affects tobacco plants in April. However, the disease did arise during high temperature in September, especially in previous occurrence of the disease and the absence of crop rotation. Incubation at low temperature and moisture decreased disease severity and adversely affected *R. solanacearum* population (Islam and Toyota, 2004).

Attention has recently been drawn to the biofumigation which uses plant VC emitted by plant residues to manage plant diseases. The process of biofumigation is known as soil bio-disinfection, and the release of antimicrobial metal ions as well as organic acids cause pathogen suppression. A variety of phytopathogens in soil including *R. solanacearum*, were successfully eradicated by biofumigation using molasses or wheat bran (Nion and Toyota, 2015).

## 5 Cultural approaches

Cultural approaches include agricultural practices that increase crop yield and quality and lessen the impact of pathogens (Ajilogba and Babalola, 2013). Crop rotation is a low-cost strategy for controlling plant pathogens that entails growing various crops on the same land during different growing seasons (Ajilogba and Babalola, 2013). Similar crops being grown repeatedly may result in the establishment of specific pathogen populations; for instance, planting tomatoes on same location every year will promote the growth of pathogens in the soil. Crop rotation reverses this negative trend and lowers the prevalence of diseases brought on by pathogens in soil (Janvier et al., 2007). It has been demonstrated that growing potatoes in rotation with other crops such as sorghum, carrots, millet increases potato production in comparison to mono-cultured tubers while reducing the bacterial wilt occurrence (Katafiire et al., 2005).

Efficient management of soil plant diseases through crop rotation can be achieved by complete removal of infested soil and replacement with healthy soil. Crop rotation has several advantageous effects such as organic matter and structural management in soil and reducing soil erosion, which is frequently brought on by mono-cropping for long time (Janvier et al., 2007). Crop rotation prevents the establishment of particular plant pathogenic populations, whereas continuous cropping with the same susceptible host plant has the opposite effect and is frequently linked to a decline in plant diseases (Janvier et al., 2007). For instance, when a susceptible tomato variety was grown after corn, lady’s fingers, cowpea, or resistant tomato, the development of bacterial wilt was postponed by 15-20 days with 30-40% reduction in overall disease severity (Nion and Toyota, 2015). Two to four times higher potato yield with 50-80% reduction in disease severity was achieved when potato crop was rotated with millet, phaseolus beans, sorghum, and maize comparing to mono-cultivation (Katafiire et al., 2005). In a case study of multi-cropping and revealed that the reduction in the severity of bacterial wilt disease is because of inhibition effect of root exudates of *Allium tuberosum* against *R. solanacearum*.

Researchers reported the reduction of in the occurrence of bacterial wilt through proper fertilizer applications. The most investigated fertilizer for preventing plant diseases is calcium. Plants with more Ca content showed reduced pathogen population and disease severity in tomato stem. In addition, a rise in Ca uptake by tomato shoots was associated with a decline in pathogen population (Nion and Toyota, 2015). The application of different fertilizers N + P + K decreased disease by 60% resulted in enhanced yield of potato. According to Hacisalihoglu et al. (2007) tomato leaves’ distribution of nutrients changed as a result of bacterial wilt infection. The combination of organic fertilizer and rock dust application managed bacterial wilt in tomato.

A number of components in plant cell walls affect how susceptible or resistant they are to pathogen infections, and silicon is regarded as being advantageous for both plants and animals. The use of silicon and chitosan together decreased the frequency of tomato BW by fostering resistance. The use of silicon fertilizer has also been shown to enhance tomato yield while lowering bacterial wilt populations and incidence (Hartmann, 2002). When compared to control soil, soil treated with farmyard manure caused significant increase in yield of tomato and reduction in BW severity. This may be because the physicochemical properties of the organically treated soil have improved, which is advantageous for crop growth.

Grafting is an asexual method of propagating plants that involves attaching pieces of different plants together so that they will eventually fuse and develop into a single plant. Consequently, a grafted plant is a component combination of different plants (Hartmann, 2002). It entails grafting a plant’s top portion of a required trait onto a disease resistant rootstock. Several vegetable crops resistant to soil pathogens including *Ralstonia* has been developed through grafting (King et al., 2008; Louws et al., 2010).

## 6 Chemical approaches

Chemical approaches include the utilization of synthetic chemicals for the control of plant diseases. Over the years, bacterial wilt has been managed using a variety of chemical techniques. However, no method is effective when used alone due to the pathogen’s complexity (Yuliar et al., 2015). To prevent bacterial wilt in ginger, methyl bromide use for soil treatment was suggested (Ishii and Aragaki, 1963). Following the methyl bromide ban, chloropicrin’s ability to lessen bacterial wilt of ginger in China was assessed. Chloropicrin covered in polyethylene film decreased *R. pseudosolanacearum* soil count and increased plant yield. The most effective rhizome protectant, oxytetracycline at 400 ppm, was reported to increase yield and decrease plant mortality against BW disease. Plant mortality rates of 29.32% and 41.26%, respectively, were found to be effective with streptocycline and oxytetracycline at lower rate. Ginger plant mortality was reported to be reduced by carbendazim and copper oxychloride but not as effective as antibiotics (Suseela Bhai et al., 2019).

These commercial chemical substances have had limited effectiveness in controlling bacterial wilt in the field. In order to manage BW economically a technology that combines soil solarization and soil improvement with CaCl has been developed (Suseela Bhai et al., 2019). The field population of *R. pseudosolanacearum* was significantly reduced in subsequent field testing of CaCl. Additional field trials in the endemic BW regions showed complete control on BW. The study’s findings can be used to develop a practical and efficient integrated control measure for the treatment of ginger’s bacterial wilt (Suseela Bhai et al., 2019). Although not always, it has been shown that using pesticides to combat BW results in a greater net benefit. Some pesticides may persist in the environment for long time, produce pollution in water and soil, and cause toxicity to consumers due to their ignorance and improper application (Dasgupta et al., 2007). Due to the negative effects on human and environmental health and the emergence of pathogen resistance, the use of chemicals like antibiotics to manage diseases has been criticized.

## 7 Nano technological approaches

Nanotechnology developed as one of the 21^st^ century’s most quickly progressing sciences. Nanotechnology offers a viable solution to the difficulties involved with the identification and control of soil pathogens, such as *R. solanacearum* (Khan et al., 2021; Guo et al., 2022). According to recent research, nanoparticles act as a possible biosensor and antibacterial agent for detecting plant infections, particularly soil-borne pathogens. Several metallic oxide nanoparticles have been investigated for their potential to act as protective agents against *R. solanacearum* (Khairy et al., 2022). NPs of ZnO, FeO, and CuO were recently investigated against the tomato bacterial wilt pathogen Ralstonia solanacearum. The findings demonstrated that NPs, particularly CuONPs, greatly decreased the disease incidence on tomato. There was also a considerable improvement in the morpho-physiological characters of plants (Jiang et al., 2021). ChNPs were found to be effective against tomato BW (Khairy et al., 2022). The maximum zone of inhibition was found in an *in vitro* testing at a dose of 200 g/ml. *In vivo* assays revealed a reduction in disease severity following foliar application of ChNPs to pathogen infected plants. The ChNPs were discovered to interact directly with the cell wall of bacteria, producing shape changes, flagella loss, and lysis. According to Santiago et al. (2019), ChNPs loaded with AgNP demonstrated antibacterial efficacy against *R. solanacearum*.

## 8 IDM approaches

In integrated disease management (IDM) various management techniques are used to achieve the highest possible level of control. The main objectives of an IDM program, in accordance with Agrios (2005), is the reduction of initial inoculum and its effectiveness, boost host resistance, boost host resistance, and postpone disease development. Because of the wide range of hosts and the complexity of the pathogen, a single management approach could not work well. But when several approaches were used in combination, the effectiveness of control enhanced up to 100% (Wu et al., 2020). To get rid of BW disease, many methods such as cultural practices, soil amendments, use of resistant cultivars and organic formulations were used in combination (Wu et al., 2020). Generally, combine use of 2-3 methods from biological, cultural or chemical approaches can reduce the BW disease by 30-90%. For example, the rate of BW in tomatoes was tracked in soil with *R. solanacearum* after adding a mixture of agricultural and organic industrial wastes along with a chemical pesticide Actigard. The disease was reduced by 29% when the organic mixture was used, and by 6% when Actigard was used. Adding the organic mixture and Actigard, on the other hand, caused 53% disease reduction. Similarly, using biocontrol agents along with their substrates like sucrose, lysine work better together to stop bacterial wilt in tomatoes. The addition of substrates makes it easier for BCAs to colonize the roots of tomatoes (Nion and Toyota, 2008).

## 9 Action mechanism of different management approaches

Different action mechanisms were reported for different management approaches used to control bacterial wilt or its pathogen *R. solanacearum*. Some approaches use direct killing or suppression of the pathogen while other approaches support indirect action mechanisms. Breakdown organic matter (OM) may directly affect the soil pathogens by producing substances that stop them from living. This limits the nutrients that are available. It could also make microorganisms work more, making competition effects more likely. Soil amended with organic matter have a direct effect on the production. They are helpful in improving soil’s chemical and physical properties, which can help plants grow (Bailey and Lazarovits, 2003; Yi et al., 2021). The soil breakdown of organic matter releasing natural chemicals with different inhibitory properties (Bailey and Lazarovits, 2003). Carbon from OM increases microbial activity in soil and makes it more likely that competition effects will happen in the soil (Bailey and Lazarovits, 2003). It has been shown that adding organic matter to soil makes microorganisms that fight pathogens work more (Akhtar and Malik, 2000). Also, OA often have molecules that are biologically active, which can affect the microbes in the soil. The increasing activities of dehydroascorbate, monodehydroascorbate, peroxidase were recently reported to enhance the host resistance against BW. It is reported that adding microorganisms to natural soil with bio-amendments worked most of the time. The effects on the soil microbial communities were very different depending on the combinations of organisms and their number and type. A new strategy to combat BW is to use a rhizosphere biofilm that only forms in organic hydroponic system (Avinash et al., 2017).

Most researchers reported that the plant residues work by killing microbes and then indirectly stopping the pathogen by making the soil properties better (Cardoso et al., 2006). The 5-(4-acetoxy-1-butynyl)-2,2’-bithienyl and 5-(3-buten-1-ynyl)-2,2’-bithienyl were found to be the antimicrobial compounds in *T. patula* that stopped *in vitro* growth of *R. solanacearum*. *Cryptomeria japonica* released ferruginol, and *Cyphomandra betacea* had a protein that inhibit glycosidase activity which stopped *in vitro R. solanacearum* growth. Lansiumamide B, which was found in *Clausena lansium* seeds stopped tobacco bacterial wilt better than the antibiotic. It found that the anti-disease effects of compost made from olive waste seemed to come from a combination of the effects of nutrients competing and antagonistic microbes. Although, it is not clear how pig slurry makes the population of *R. solanacearum* go down faster and stops diseases from spreading, but shifts in the bacterial community profiles have been suggested as a possible explanation.

Another study revealed that the way farmyard manure and poultry stopped BW was linked to the activities of a greater number of fungi and bacteria (Islam and Toyota, 2004). The pathogen suppression was linked to the decreased disease severity in this study. In pot evaluation, organic acids, amino acids, and sugars were tested to see how well they stopped bacterial wilt in tomatoes. When lysine was added to a pumice medium and soil, there was a higher drop in tomato wilt (Nion and Toyota, 2008). This wasn’t because of the ISR, but because of changes in the microbial community in soil that made the pathogen die more quickly. Riboflavin, on the other hand, caused a number of defense mechanisms in cell suspensions which made tobacco resistant to R. solanacearum. Aminobutyric acid also made polyphenol activity go up and catalase activity go down in tomato plants. This suggests that aminobutyric acid made the tomato plants resistant to bacterial wilt. Methyl gallate was also reported to kill *R. solanacearum* very well (Fan et al., 2014).

*Enterobacter cloacae* stopped bacterial wilt disease in potatoes and raised yields by using antibiosis and inducing plant systemic resistance. The effective control of BW by *P. aeruginosa* and *P. syringae* was also attributed to mechanism of antibiosis (Mohammed et al., 2020). In another study, the screening of a large number of endophytic isolates showed the antibiosis activity of *S. warneri* and *B. velezensis* against *R. solanacearum* (Agarwal et al., 2020). Through a process called niche exclusion, *B. amyloliquefaciens* manage tobacco BW very well. The combine effect of niche exclusion, gene copy number reduction in the rhizosphere soil, and direct antagonism were reported for BW disease suppression of *B. amyloliquefaciens*. Production of antibacterial volatile compounds that act as plant growth inducer as well as have direct killing effect against *R. solanacearum*were also reported. According to research by Tahir et al. (2017), VOCs produced by *B. amyloliquefaciens* and *B. artrophaeus* were found to kill *R. solanacearum* while also promoting plant growth. By enhancing the improvement in soil’s properties soil amendment with *B. amyloliquefaciens* and marigold powder together reduced the prevalence of tobacco bacterial wilt disease. The action mechanism responsible for physical approaches mainly includes the use of high or low temperature. The investigation on finding the action mechanism of soil solarization in reducing BW disease revealed that soil solarization caused reduction in K, Na, zinc and overall pH which ultimately affect the survival of *R. solanacearum* (Baptista et al., 2006).

## 10 Future prospects and challenges

Cultural practices have an impact on soil characteristics, which affect the distribution and viability of pathogens. More studies are being done to determine how these practices affect microbial communities or how effective they are at preventing the spread of pathogens in soil. Risk forecasting and technical guidance may benefit greatly from indicators of soil health.

Preventive measures are important for keeping fields free of soil pathogens. *R. solanacearum* can live in plant materials, water, and soil for extended periods of time. Thus, in order to boost agricultural output by preventing this disease, it is important to disinfect seeds, soil, water.

It is necessary to examine the pathogen’s dynamics and genetic diversity in order to understand the influence of geographic and environmental variables on the population structure of *Ralstonia* spp.

The understanding of virulence mechanisms of pathogen, complex regulatory networks, and pathogenicity determinants should be advanced through research and analysis.

Early identification of *R. solanacearum* in soil and water is important for avoiding its spread to new locations. Recently, a sensitive quantitative technique for detecting *Ralstonia solanacearum* in soil based on the most probable number analysis of PCR findings was developed. This technique allows the detection of pathogens at very low concentrations.

Complicated interactions occur between soil-borne pathogens such as *R. solanacearum* and plants, involving both abiotic and biotic and variables. Prior research focused mostly on the biotic variables that regulate BW. Future investigations should also be focused on abiotic factors that help in reducing bacterial population.

Researchers have treated bacterial wilt a variety of management strategies; however, few studies, particularly economic assessments, have assessed the efficacy of these methods to increase crop output.

To be able to forecast outbreaks of bacterial wilt, it will be necessary to do more research to determine the severity of crop damage caused by *Ralstonia* spp. and to understand the disease’s epidemiology.

## Author contributions

ZW and SC: Conceptualization, Literature survey, Writing major original draft. WL and HZ: Writing- review and editing. JZ and ZZ: Figure designing, Tabulation, Writing- review and editing. All authors contributed to the article and approved the submitted version.
